# Phenotypic and genotypic detection of methicillin-resistant *Staphylococcus aureus* in hunting dogs in Maiduguri metropolitan, Borno State, Nigeria

**DOI:** 10.14202/vetworld.2016.501-506

**Published:** 2016-05-24

**Authors:** Muhammad Mustapha, Yachilla Maryam Bukar-Kolo, Yaqub Ahmed Geidam, Isa Adamu Gulani

**Affiliations:** Department of Veterinary Medicine, Faculty of Veterinary Medicine, University of Maiduguri, PMB 1069 Maiduguri, Borno State, Nigeria

**Keywords:** dogs, genotypic, methicillin, phenotypic, *Staphylococcus aureus*

## Abstract

**Aim:**

To determine the presence of MRSA in hunting dogs in Maiduguri metropolitan.

**Materials and Methods:**

Phenotypic methods used includes microscopic technique, colony morphology study, catalase-coagulase tests, and the use of mannitol salt agar test, oxacillin resistance screening agar base, and antibiotic susceptibility testing methods. Genotypic approach was used for deoxyribonucleic acid extraction, and the presence of *nuc* and *mecA* gene was detected using polymerase chain reaction (PCR) techniques.

**Results:**

Examination of 416 swab samples from nasal and perineal region of dogs revealed a total of 79.5% of *S. aureus*, where 62.5% of the isolates were MRSA. Molecular analysis revealed that 7*nuc* genes specific for *S. aureus* from 20 presumptive MRSA assay were all *mecA* PCR negative. The isolates were sensitive to gentamicin and ciprofloxacin but proved resistant to cefoxitin and oxacillin.

**Conclusion:**

High isolation rate of MRSA was found in hunting dogs. Significant level (p<0.05) of MRSA was isolated in the nasal cavity of hunting dogs than its perineum. Only *nuc* genes were detected from the MRSA isolates.

## Introduction

Staphylococcal infections are of major importance in both Human and Veterinary Medicine. *Staphylococcus aureus* is a major resident or transient colonizer of the skin and the mucosa of humans and primates [1]. *S. aureus* is occasionally found on domestic animals, although other species of staphylococci predominate. *S.aureus* produces an extracellular thermostable nuclease, encoded by *nuc* gene [2].

*S. aureus* has characteristic ability to rapidly develop resistance to virtually any antibiotics coming into clinical use [3]. Resistance to methicillin that indicated resistance to all beta-lactam agents was first reported in 1961, which marked the appearance of methicillin-resistant *S. aureus* (MRSA) [4]. MRSA is becoming a major public health concern because companion animals often are in close physical contact (touching, petting, and licking) with their owners, exposing them to infection with pathogenic bacteria [5]. Dogs are usually colonized by MRSA strains from humans [6,7].

When *S. aureus* gains entry into the host, it is able to cause a variety of infections from mild skin infection to life-threatening invasive infections such as brain abscesses, endocarditis, pericarditis, pneumonia, arthritis, osteomyelitis, urinary tract infection, and toxic shock syndrome [1]. The report above therefore, indicates that hunting dogs could be colonized by MRSA. Colonization of MRSA in dogs has been extensively studied in Europe and most parts of the world. However, in Nigeria, there is very little documented information on (MRSA) colonization of dogs.

There is the presence of MRSA in hunting dogs in Maiduguri Borno State, which is evident as a result of the presence of *mecA* polymerase chain reaction (PCR) negative *S. aureus* in the isolate. Therefore, the objective of this research is to determine the presence of MRSA, antibiotic sensitivity pattern of MRSA isolates, and the presence of *nuc* and *mecA* gene from MRSA isolates using phenotypic and genotypic techniques.

## Materials and Methods

### Ethical approval

This research was approved by the Faculty of Veterinary Medicine Ethics and Research Committee, University of Maiduguri, Borno State, Nigeria.

### Study area

**Figure-1:**
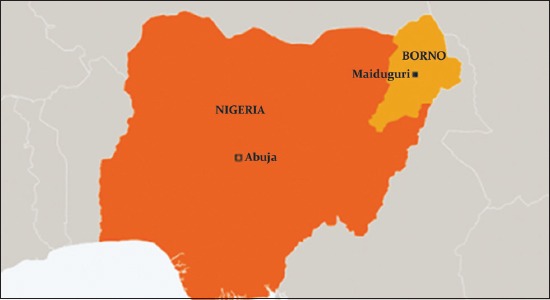
Map of Nigeria showing Maiduguri metropolitan in Borno State (study area).

Maiduguri is the capital and the largest urban center of Borno State, North Eastern Nigeria (Figure-1). The state lies between latitude 11°32′ North and 11°40′ North and latitude 13°20′ East and 13°25′ East between the Sudan Savanna and Sahel Savanna vegetation zones, characterized by short rainy season of 3-4 months (June-September) followed by a prolonged dry season of more than 8 months duration [8].

### Sample collection

Total of 416 swab samples were collected from the four major hunting rendezvous in Maiduguri metropolitan. 211 and 205 swab samples from the nostril and perineum of the hunting dogs were analyzed. Cotton swab sticks were used to collect all the samples according to laboratory standard [9]. Each sample was labeled with an identification number and date of collection. The samples were kept on an ice pack and transported to the research laboratory, Department of Veterinary Medicine, Faculty of Veterinary Medicine, University of Maiduguri.

### Hunting dogs

Dogs were sampled with owners consent. Cotton-tipped sterile swab (Everson Industries Limited, Nigeria) was inserted into one nostril of each dog where sample was taken from the nasal mucosa and immediately inserted into the aseptic tube. The tubes were labeled and dates of collection noted. Another sterile cotton-tipped swab is rolled on the perineal region and then immediately inserted back into its tube [9].

### Isolation and identification

Enriched solid medium of 5% blood agar (Sigma^®^ Switzerland) was prepared according to the manufacturer’s instruction and inoculated with the swabs [10]. Sterile wire loop was used to streak the inoculums to get discrete colonies. The inoculated plates were incubated aerobically at 37°C for 24 h and observed for yellowish-white colonies with smooth slightly raised surfaces. Some positive colonies have complete zones of hemolysis while others were non-hemolytic.

### Colony morphology

Gram-stain of the collected samples was performed to identify staphylococci by their gram reaction. Samples that were cocci arranged in grape-like clusters were subjected to catalase and coagulase tests. The positive isolates were streaked on mannitol salt agar (MSA, Oxoid) which is a selective medium for *S. aureus*, and plates were incubated under an aerobic condition at 37°C for 24 h. The appearance of yellowish colonies on MSA was presumed to be *S. aureus*.

### Oxacillin resistance screening agar base (ORSAB)

ORSAB is a medium for the screening of MRSA, the medium is nutritious, selective and contains peptones for the growth of microorganisms. The medium has high salt and lithium chloride concentration to suppress non-staphylococcal growth, with mannitol and aniline blue, for the detection of mannitol fermentation. The antibiotics contained in ORSAB selective supplement are oxacillin at 2 mg/L to inhibit methicillin-sensitive *S. aureus*, and polymyxin B for the suppression of other bacteria that are able to grow at such a high salt concentration,e.g. *Proteus* spp. typical colonies of MRSA are intense blue on a colorless background enabling the organism to be more easily identified in mixed culture than the pale yellow colonies seen on MSA.

### Antibiotic susceptibility testing

The ATS of MRSA isolates was determined according to the method of Bauer-Kirby [11]. Using the commercially prepared disc (Oxoid, UK) with a known concentration of antibiotics, freshly sub-cultured MRSA and well-isolated colonies from ORSAB plates were emulsified in 3-4 ml of sterile normal saline. The turbidity of the suspension was adjusted to the turbidity of standard equivalent to 0.5 McFarland [12]. Mueller-Hinton agar medium was prepared, and a sterile cotton swab stick was dipped into the suspension. Excess fluid was removed by pressing and rotating the swab against the side of the tube above the suspension. The dried surface of the Mueller-Hinton agar was inoculated by streaking the swab evenly over the surface of the medium in three directions, rotating the plate approximately 60° to ensure even distribution [9]. Five antimicrobial discs were dispensed into each inoculated plates and incubated at 35°C for 24 h. Zone of inhibition were measured in millimeters (mm) using vernier caliper. The sizes of the zones of inhibition were interpreted according to CLSI [12] criteria. The following 10 antibiotics were tested FOX 30 ug, DA 2 ug, SXT 25 ug, CIP 5 ug, E 15 ug, KZ 30 ug, C 30 ug, CN 10 ug, TE 30 ug, and OX 1 g (Oxoid, UK). For the interpretation of susceptibility toward oxacillin disc, growth within the zone of inhibition was considered indicative of methicillin resistance. According to the classification criteria given by CLSI [12], a diameter of inhibition zones of ≤10, 11-12, and ≥13 by 1 ug of oxacillin is categorized as resistant (R), intermediate (I), or susceptible (S) to oxacillin accordingly. For cefoxitin disc, a diameter of inhibition zones of ≤24 and ≥25 mm correspond to the class of staphylococci considered as resistance or susceptible for oxacillin, accordingly. There is no intermediate category of classification for staphylococci using the cefoxitin disc diffusion test [12].

### Genotypic characterization

A total of 80 phenotypically detected MRSA from hunting dogs, 20 samples from this number were randomly selected for the genotypic analysis using PCR for the detection of *S. aureus* specific gene (*nuc* gene), and the *mecA* gene encoding the resistance as described by Perez-Roth *et al*. [13].

### Deoxyribonucleic acid (DNA) extraction

A loop full MRSA isolates were scooped into 1.5 ml tube that contained 400 μl of lysis buffer and 4 μl proteinase k and was vortexed (Fisher brand, Allied Fisher Scientific, USA), for 2 min to get a homogeneous mixture. This was followed by incubation at 55°C for 3 h in the thermocycler (Strata gene, USA). 400 μl of phenol/chloroform (PC) was added to the tube and mixed gently for 1min. It was then spun in a microcentrifuge for 10 min at maximum speed (10,000 rpm). The supernatant was transferred to another tube, and 400 μl of PC was added, vortexed, and then it was spun for 10 min at 10,000 rpm. The supernatant was transferred to a new tube, 300 μl of chloroform was added and vortexed, spun for 1 min at 10,000 rpm, and the supernatant was also transferred to another tube. 825 μl of 100% ethanol and 25 μl of sodium acetate were added and incubated in the freezer overnight.

The following day, the samples were centrifuged using high speed refrigerated centrifuge (Harvey Instruments Inc. USA) for 20 min and the supernatant was discarded. One (1 ml) of 70% ethanol was added to the samples, mixed and centrifuged for 20 min at 13500 rpm; the supernatant was discarded and dry spun for 1 min, and the residual ethanol was removed. The DNA pellets were allowed to dry at room temperature. The pellets were resuspended in 20 μl of water. After the DNA extraction, 10 μl of loading dye (bromophenol blue) was mixed with 5 μl of the DNA pellets and pipetted into the wells of the gel. Finally, electrophoresis was carried out to determine the presence of DNA.

### PCR primers dilution

Primers that corresponded to *nuc* gene specific for *S. aureus* and *mecA* gene were obtained from Integrated DNA Technologies, USA. The primers were resuspended in sterile distilled water, and the diluted primers were stored at −20°C.

### Detection of nuc and mecA gene by PCR

The PCR amplification was done according to Perez-Roth *et al*. [13]. Using the following primers that will detect the *nuc* gene in *S. aureus* and *mecA* gene with the amplicon size of 276 bp and 533 bp; The *nuc* primers were 5’-GCG ATT GAT GGT GAT ACG GTT-3’ and 5’-AGC CAA GCC TTG ACG AAC TAA AGC-3’, whereas the *mecA* primers were 5’-AAA ATC GAT GGT AAA GGT TGG C-3’ and 5’-AGT TCT GCA GTA CCG GAT TTG C-3’. The PCR amplification mixture consisted of 10 µl of PCR premix, 2 µl DNA templates, 2 µl primers, and 16 µl of distilled water. A total of 40 cycles were used to amplify 533 bp of *mecA* gene and 276 bp of *nuc* gene specific for *S. aureus*. DNA pre-denaturation occurs at 94°C for 5 min and DNA denaturation at 30 s to 1 min primers annealing at 55°C for 30 s. Approximately 5°C below primers temperature, extension of the two strands at 72°C for 60 s and a final extension at 72°C for 4 min. 10 µl each of the PCR products for *mecA* and *nuc* gene were analyzed separately on 2% agarose gel (Biogene, UK). Electrophoresis was performed in TBE buffer at 180 V for 1 h, and the gel was subsequently stained with 3 µl of ethidium bromide (Sigma, UK). DNA bands were visualized using UV-light with the camera (Gel Doc 2000, Bio-Rad) and photographed.

### Data analysis

Fisher’s exact test (Graph pad^®^Software Inc.) was used to determine the probability and significance of MRSA detection from the skin, perineal region, and nasal cavity of hunting dogs. The values were considered significant (p< 0.05).

## Results

**Figure-2:**
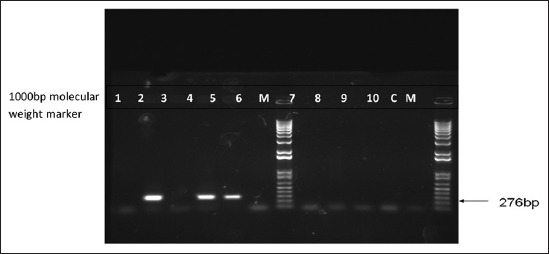
Agarose gel electrophoresis of polymerase chain reaction of methicillin-resistance *Staphylococcus aureus* isolate. Lanes 2, 4, and 5 are positive for nuc gene as indicated by 276 bp.Lane 1, 3, 6, 7, 8, 9, and 10 are negative samples. Lane M is the molecular weightmarker. Lane C is the negative control.

**Figure-3:**
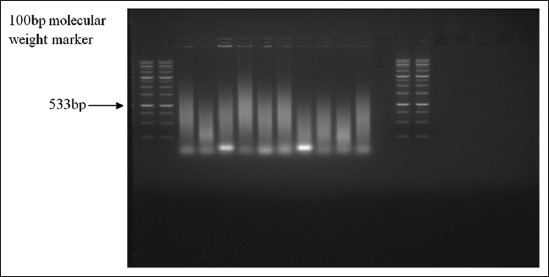
Agarose gel electrophoresis pattern of polymerase chain reaction of methicillin-resistance *Staphylococcus aureus* isolate. Lane M: Molecular weight marker. Lane 1-10: Negative for mecA. Lane C: Negative control.

Total of 416 swab samples from the nostril and perineal region of hunting dogs of *S*. *aureus* revealed 128 (79.5%) positive isolates. Microscopic examination of Gram-stained colonies showed Gram-positive cocci arranged in irregular grape-like clusters, some appearing in single, whereas others in pairs, short chain, or tetrads. Results of catalase and coagulase positive isolates are 141 (87.6) and 128 (79.5%), respectively. Table-1 shows results of Gram-staining, biochemical test and ORSAB screening of staphylococcal isolates in hunting dogs in Maiduguri metropolitan, Borno State. The values of *S. aureus* isolated from the nostril and perineum of hunting dogs were 78 (36.9%) and 50 (24.4%) respectively. The values of MRSA isolated from the nostril and perineum of hunting dogs were 50 (23.7%) and 30 (14.7%), respectively (Table-2). The result of antimicrobial susceptibility test indicated that the isolates were highly resistant to FOX (99%), OX (88.7%), TE (73.4%), and KZ (70.0%), while they were highly susceptible to CN (98%), CIP (90.8%), C (87.6%), SXT (87.8%), E (81.6%), and DA(79.6%) as presented in Table-3. Multidrug Resistance (MDR) Profile of MRSA isolated from hunting dogs in Maiduguri Metropolitan is shown in Table-4. 20 presumptive MRSA isolates assayed 7 bands showed evidence expression of *nuc* gene specific for *S. aureus* with a molecular weight of 276 bp which is presented in Figure-2. This confirms the assumption based on the phenotypic detection that some of the strains were *S. aureus*. The result of PCR based on targeted *mecA* gene revealed that none of the isolates possessed *mecA* gene as represented in Figure-3.

**Table-1:** Gram-staining, biochemical test and ORSAB screening of *S. aureus* isolates in hunting dogs in Maiduguri metropolitan, Borno State.

Test	Result of positive isolates (%)	Result of negative isolates (%)	Total
Gram reaction	161 (38.7^a^) S	255 (61.3^b^)	416
Catalase test	141 (87.6^c^)	20 (12.4^d^)	161
Coagulase test	128 (79.5)	33 (20.5)	161
ORSAB screening	80 (62.5^k^)	48 (37.5^y^)	128

Figures in brackets are percentage occurrence of staphylococcal isolate species. Values denoted by different superscripts for a given parameter are significantly different (p<0.05). ORSAB=Oxacillin resistance screening agar base, *S. aureus*=*Staphylococcus aureus*

**Table-2:** Percentage distribution of *S. aureus* and MRSA isolated from hunting dogs in Maiduguri metropolitan.

Source	Site	*S. aureus* (-ve)	*S. aureus* (+ve)	MRSA (+ve)	Number of samples
Dog	Nasal	133	78 (36.9)	50 (23.7)	211
	Perineum	155	50 (24.4)	30 (14.7)	205
	Total	288	128 (30.8)	80 (19.2)	416

MRSA=Methicillin-resistance *Staphylococcus aureus*, *S. aureus*=*Staphylococcus aureus*

**Table-3:** Antibiotic susceptibility pattern of MRSA isolated from hunting dogs in Maiduguri metropolitan, Borno State.

Susceptibility pattern			

Antibiotics	Resistant number of isolates (%)	Intermediate number of isolates (%)	Susceptible number of isolates (%)
Cefoxitin	97 (99.0)	0 (0.0)	1 (1.0)
Cefazolin	69 (70.0)	6 (6.1)	23 (23.5)
Chloramphenicol	8 (8.2)	4 (4.1)	86 (87.8)
Ciprofloxacin	3 (3.1)	6 (6.1)	89 (90.8)
Clindamycin	15 (15.3)	5 (5.1)	78 (79.6)
Erythromycin	14 (14.3)	4 (4.1)	80 (81.6)
Gentamicin	1 (1.0)	1 (1.0)	96 (98.9)
Oxacillin	87 (88.8)	1 (1.0)	10 (10.2)
Tetracycline	72 (73.4)	4 (4.1)	22 (22.4)
Sulfa/Trimethoprim	7 (7.1)	5 (5.1)	86 (87.8)

MRSA=Methicillin-resistance *Staphylococcus aureus*

**Table-4:** Multidrug resistance profile of MRSA isolated from hunting dogs in Maiduguri metropolitan.

Number of drugs resisted (%)
0 (0.00)
1 (2.04)
2 (6.12)
3 (34.7)
4 (35.7)
≥5 (21.4)

MRSA=Methicillin-resistance *Staphylococcus aureus*

## Discussion

Investigations on phenotypic detection of MRSA in hunting dogs in Maiduguri Metropolitan revealed higher isolation rates (19.2%) of these pathogens in dogs compared to the findings of Loeffler *et al*. [14], Kottler *et al*. [15], and Chah *et al*. [16] who, respectively, recorded 9.0%, 3.3%, and 12.8% of MRSA in dogs in the UK, the US, and Nigeria.

This may probably be attributed to variation in the breeds of dogs used in this finding, indiscriminate antibiotic therapy, harsher environmental challenges commonly encountered during hunting and could also be associated to starvation which results in stress that affects dog’s immunity. Kutdang et al. [17] supported the assertion above from his finding that hunting dogs are more exposed to the outside environment and hence stood the chance of contracting more infections than household dogs. Floras et al. [18] further augmented the findings of this study with a report of an increasingly higher detection rate of MRSA in dogs in Canada.

The distribution of *S. aureus* and MRSA recorded from the nasal cavity of hunting dogs was significantly different (higher) than the values recorded from the perineal region of dogs. These suggest that higher possibilities of contamination rate of dogs are through the nostril. Contaminations can also result through scavenging of death carcasses exposed to antibiotics from backyard poultry operations. These tallies, with the findings of Rich and Roberts [19] and Khanna *et al*. [20] who reported that MRSA colonization in animals, are better detected through nasal sampling. Rich and Roberts [19] further confirmed this through a record of a single case of MRSA detection from nasal swab samples of 255 dogs and not from the throat and skin of the same animals.

Antimicrobial susceptibility pattern of the MRSA isolates has also been investigated indicating a high level of resistance to FOX and OX. This finding, therefore, implies that FOX based assays are particularly important for low-level OX resistant MRSA detection [21]. Phenotypic antimicrobial susceptibility test needs particular care when using OX as a test substrate because of heterogeneous *in vitro* expression of Methicillin-resistance (hetero-resistance) in nearly all of currently disseminated MRSA clonal lineages. Heteroresistance can be either detected using high inocula as recommended by the Clinical Laboratory Standard Institutes, or by use of FOX disks since this antibiotic is less affected by heterogeneous expression [12].

The MRSA isolates were highly susceptible to CIP and CN. CIP, a member of the fluoroquinolones that are newer drugs with mode of action on DNA inhibition and are relatively expensive and less available for abuses [22]. In addition; CN, an aminoglycoside, also showed high activity against MRSA, which may be as a result of the complexity of the aminoglycoside and the route of administration [22].

It was concluded that 59 out of 80 isolates of MRSA were multidrug resistant (MDR) in this study. This finding was supported by Abeer *et al*. [23], who reported that 14 out of 51 MRSA isolates were MDR. Methicillin-resistant coagulase negative staphylococci (MRCoNS) from healthy dogs in Nsukka, Nigeria, isolated revealed that 13 out of 109 isolates of MRCoNS were MDR [16].

The PCR analysis did not reveal any *mecA* positive samples, and this might indicate the presence of *mecA* PCR negative MRSA isolates in Maiduguri. A previous study by Garcia-Alvarez *et al*. [24] had reported a novel allele of the *mecA* gene encoding an alternative penicillin binding protein that mediates methicillin resistance among bovine *S. aureus* isolates, and humans in the UK, Denmark, and Germany that were Methicillin-resistant but *mecA* PCR negative.

These novel alleles of the *mecA* gene (*mecA*_LGA251_) have 70% nucleotide identity to the archetypal *mecA* gene. Moreover, the findings highlight the possibility that additional *mecA* allele is in circulation in the environment, and therefore, could be acquired by *S. aureus* and leads to the emergence of new MRSA strain. However, antimicrobial susceptibility testing and other routine culture will identify *S. aureus* isolates encoding *mecA*_LGA251_ as methicillin-resistant [24]. Furthermore, Kriegeskorte *et al*. [25] also found MRSA isolates with novel genetic homolog among human in a study of human MRSA isolates in Germany.

The results of the PCR analysis in the current study which revealed *nuc* genes at 276 bp which tallied with the findings recorded by Merlino *et al*. [26], Saiful *et al*. [27], and Szczepanik *et al*. [28], who used modified PCR analysis (multiplex PCR) for the detection of *mecA* and *nuc* genes in multidrug resistance and non-multidrug resistance MRSA.

## Conclusion

Conclusively, MRSA was phenotypically detected with significant (p<0.05) isolation rate in the hunting dogs. Microbiological and PCR results confirm the presence of MRSA in hunting dogs in Maiduguri Metropolitan, Borno State, Nigeria. Higher percentages (50.0%) of MRSA were detected from the nasal cavities of dogs than the perineal region (30.0%).

## Author’s Contributions

This study was conceived by MM, YMB, and YAG. MM collected the data during field work. The data were compiled by MM, analyzed by MM and IAG. Write up by MM. All authors read and approved the final manuscript.
